# Edaphic heterogeneity and the evolutionary trajectory of Amazonian plant communities

**DOI:** 10.1002/ece3.8477

**Published:** 2021-12-20

**Authors:** Samuli Lehtonen, Robert Muscarella, Gabriel Moulatlet, Henrik Balslev, Hanna Tuomisto

**Affiliations:** ^1^ Biodiversity Unit University of Turku Turku Finland; ^2^ Plant Ecology and Evolution Evolutionary Biology Center Uppsala University Uppsala Sweden; ^3^ Facultad de Ciencias de la Tierra y Agua Universidad Regional Amazónica Ikiam Tena Ecuador; ^4^ Section for Ecoinformatics & Biodiversity Department of Bioscience Aarhus University Aarhus Denmark; ^5^ Department of Biology University of Turku Turku Finland

**Keywords:** Amazonia, Arecaceae, beta diversity, community phylogenetics, edaphic gradients, ferns, Nauta/Içá Formation, Pebas/Solimões Formation, soil variation, species richness, *terra firme*

## Abstract

We investigated how the phylogenetic structure of Amazonian plant communities varies along an edaphic gradient within the non‐inundated forests. Forty localities were sampled on three terrain types representing two kinds of soil: clayey soils of a high base cation concentration derived from the Solimões formation, and loamy soils with lower base cation concentration derived from the Içá formation and alluvial terraces. Phylogenetic community metrics were calculated for each locality for ferns and palms both with ferns as one group and for each of three fern clades with a crown group age comparable to that of palms. Palm and fern communities showed significant and contrasting phylogenetic signals along the soil gradient. Fern species richness increased but standard effect size of mean pairwise distance (SES.MPD) and variation of pairwise distances (VPD) decreased with increasing soil base cation concentration. In contrast, palm communities were more species rich on less cation‐rich soils and their SES.MPD increased with soil base cation concentration. Species turnover between the communities reflected the soil gradient slightly better when based on species occurrences than when phylogenetic distances between the species were considered. Each of the three fern subclades behaved differently from each other and from the entire fern clade. The fern clade whose phylogenetic patterns were most similar to those of palms also resembled palms in being most species‐rich on cation‐poor soils. The phylogenetic structuring of local plant communities varies along a soil base cation concentration gradient within non‐inundated Amazonian rain forests. Lineages can show either similar or different phylogenetic community structure patterns and evolutionary trajectories, and we suggest this to be linked to their environmental adaptations. Consequently, geological heterogeneity can be expected to translate into a potentially highly diverse set of evolutionarily distinct community assembly pathways in Amazonia and elsewhere.

## Graphical abstract

We investigated the phylogenetic structure of Amazonian fern and palm communities on edaphically distinct sites. We show that phylogenetic community structure varies along a soil base cation concentration gradient within non‐inundated Amazonian rain forests. Our results suggest that geological heterogeneity can translate into a potentially highly diverse set of evolutionarily distinct community assembly pathways in Amazonia.

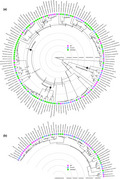

## 1 INTRODUCTION

Floristic composition of Amazonian forests varies along both edaphic and climatic gradients (Baldeck et al., 2016; ter Steege et al., 2006; Tuomisto et al., 2019). Local edaphic properties are determined by sedimentological and erosional history and can vary abruptly, thus creating a mosaic of floristically and functionally distinct units (Higgins et al., 2011; Malhado et al., 2013; Tuomisto et al., 1995). This heterogeneity has been recognized as an important factor maintaining high Amazonian diversity and has been suggested to act as a potential driver of speciation (Fine et al., 2005, 2013; Hoorn et al., 2010; ter Steege, 2009, 2006; Tuomisto, 2006, 2007). However, it remains unknown to what degree floristic variation reflects independent phylogenetic histories of the floras inhabiting edaphically distinct units.

In recent years, the relationships between ecological and historical determinants of community structure have been increasingly investigated using community phylogenetic approaches (Elliott et al., 2016; Vamosi et al., 2009). In the Amazonian context, phylogenetic structure of communities has repeatedly been shown to be related to soil variables, thus indicating that edaphic variation plays a significant evolutionary role in Amazonia. For example, flooding is an important factor that has a strong association with the phylogenetic structure of palm and tree communities in Amazonia, due to the strong filtering effect of inundation (Muscarella et al., 2019; Umaña et al., 2012). Even within the non‐inundated *terra firme* areas, western Amazonian tree communities have been found to have different phylogenetic structures on white‐sand soils and on clay soils (Fine & Kembel, 2011). Likewise, Amazonian palm communities appear phylogenetically structured along soil gradients, such as soil fertility (Eiserhardt et al., 2013; Muscarella et al., 2019).

While most studies have compared ecologically clearly contrasting communities, Lehtonen et al. (2015) and Muscarella et al. (2019) studied the phylogenetic community structure of fern and palm communities, respectively, along soil fertility gradients within non‐inundated *terra firme* forests. Both studies reported clear trends within their respective plant group, but between the plant groups the trends were contrasting. Specifically, fern communities were more species rich and composed of phylogenetically more closely related species on more fertile soils (Lehtonen et al., 2015), whereas palm species richness was higher on poor soils, where the communities also consisted of phylogenetically more clustered sets of specialist species (Muscarella et al., 2019). However, interpretation of the patterns observed by Lehtonen et al. (2015) was confounded by the spatial setup of sampling: all poor‐soil communities were located in Central Amazonia in Brazil whereas most rich‐soil communities were in Panama. Nonetheless, together these studies suggest that environmental gradients within *terra firme* forests can drive evolutionary responses strongly enough to generate communities with distinct phylogenetic patterns.

A dramatic example of edaphically determined floristic turnover zone in Amazonia stretches over 1000 km in western Brazil (Higgins et al., 2011; Tuomisto et al., 2016, 2019). To the west of this north‐south oriented boundary, soils are mostly clayey and derived from the cation‐rich Solimões formation, which corresponds to the Pebas formation in Peru. The sediments of the Solimões/Pebas formation were deposited in the Miocene under semi‐marine or lacustrine conditions (the Pebas lake system; Hoorn & Wesselingh, 2010). To the east of the boundary, more coarse‐grained and cation‐poor soils prevail that have been associated with the Içá formation (Higgins et al., 2011; Schobbenhaus et al., 2004). These soils have similar properties than soils derived from the Nauta formation in Peru, which was formed under high‐energy deltaic or fluvial sedimentary systems in the Pliocene‐Pleistocene. In addition, there are fluvial terraces along the Juruá river that are probably of Pleistocene‐Holocene age and have soils relatively similar to those of the Içá/Nauta formation.

**FIGURE 1:**
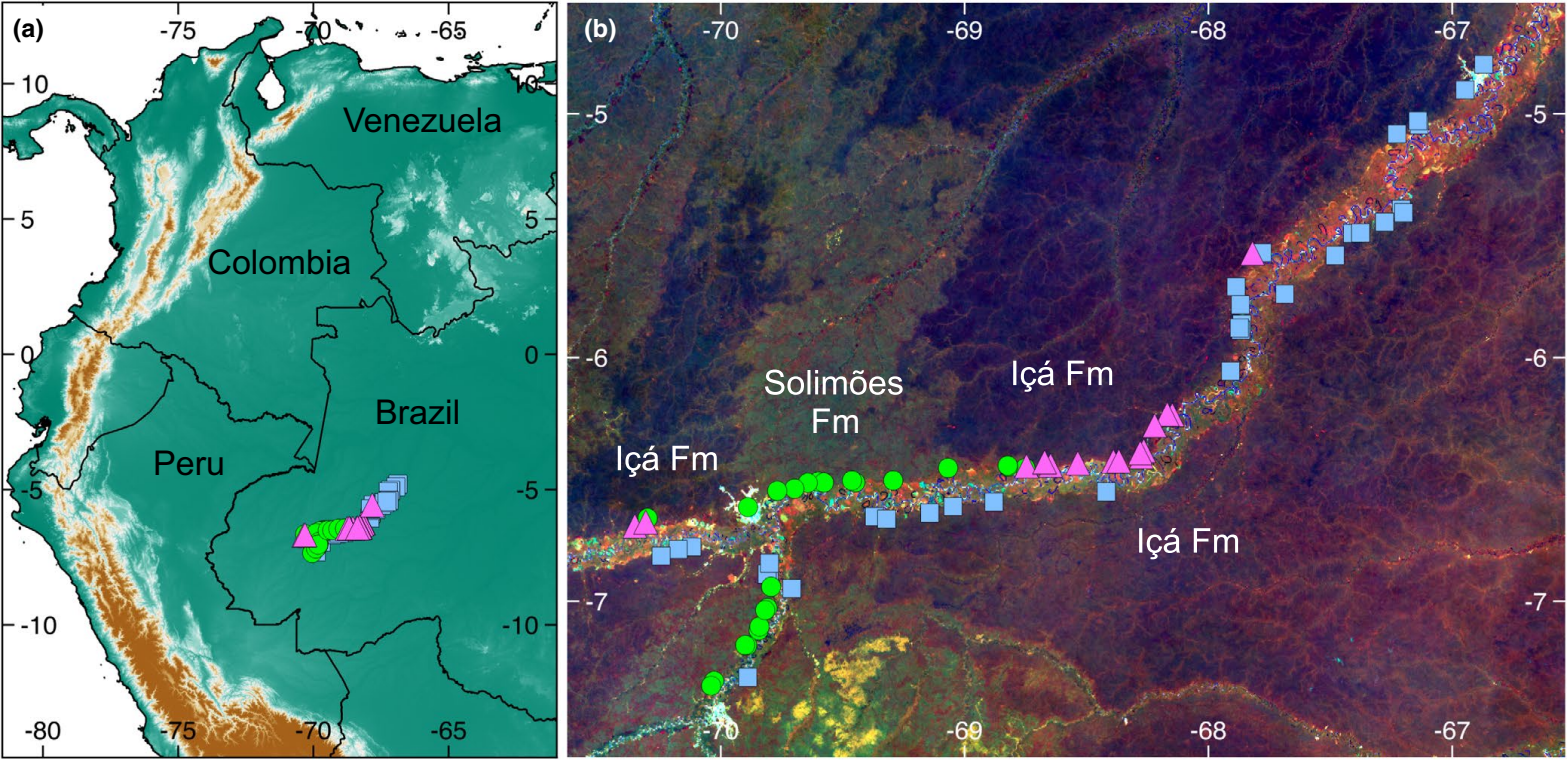
Distribution of the 40 inventory transects. Green circles correspond to transects on the Solimões Formation, blue squares to those on river terraces, and pink triangles to those on the Içá Formation. (a) Location of the study area in northwestern South America. Background map is based on the SRTM (Shuttle Radar Topography Mission) elevation model. (b) Locations of the transects within the study area along the Juruá river. Background map is a detail of the Amazon‐wide Landsat TM/ETM+ composite published in Figure 1 of Tuomisto et al. (2019)

In an earlier study, four phylogenetically unrelated plant groups that had been sampled along the Juruá river (pteridophytes, palms, Melastomataceae, and Zingiberales) showed congruent species turnover patterns between the geological formations, but there was little evidence of differentiation across the river itself along the sampled 500‐km stretch (Tuomisto et al., 2016). It was concluded that the boundary acts as an environmental filter that sorts species according to their edaphic preferences (Tuomisto et al., 2016). An intriguing question is what role such abrupt edaphic gradients may have played in the evolution of the adjacent but taxonomically distinct floras. Here, we address this question by studying how the phylogenetic structure of local palm and fern communities varies in relation to the geological boundaries (Figure 1).

We test four hypotheses. Firstly, we assess whether the floristic turnover at the Solimões‐Içá boundary is reflected also in the phylogenetic structure of local plant communities. If this is the case, it suggests that the edaphic variation maintains distinct selective landscapes in Amazonia. In this context, we also explore the relative explanatory performance of species‐level and phylogeny‐aware compositional turnover (β‐diversity) metrics in describing local variation of floristic patterns.

Secondly, in a previous study, we speculated that niche shifts in ferns might be more common from cation‐poor to cation‐rich soils than vice versa (Lehtonen et al., 2015). It might be that adaptation to cation‐poor substrates is difficult for ferns, and maybe for plants in general, due to physiological constraints (Tuomisto et al., 2014). If this is the case, the rich‐soil communities should have a more regular phylogenetic structure than poor‐soil communities: species adapted to the former could have evolved anywhere in the phylogeny, whereas species of the latter would be more concentrated to a few lineages where evolution of the necessary adaptations has been followed by in situ speciation. On the basis of Muscarella et al. (2019), we expect that palm communities show phylogenetic clustering on poor soils.

Thirdly, comparison of earlier studies suggests that fern and palm communities have contrasting phylogenetic responses to the edaphic gradients in Amazonia (Lehtonen et al., 2015; Muscarella et al., 2019). However, the interpretation of this finding is problematic given that lineage age affects the observed phylogenetic patterns of community structure (Cavender‐Bares et al., 2006; Elliott et al., 2016; Graham et al., 2018) and ferns have a much longer evolutionary history than palms do. In order to take this into account when comparing phylogenetic community patterns, we will separately analyze three fern subclades whose crown ages are roughly equal to the crown age of palms.

Finally, there are two alternative scenarios for congruence between lineages. An individualistic scenario suggests that each evolutionary lineage has its own fate, with the diversification pattern of each lineage following its own (ultimately random) trajectory. A congruence scenario suggests that when ecological conditions are similar, lineages are pushed into similar diversification pathways, in which case we would expect lineages of comparable phylogenetic depth and ecological background to produce more similar community phylogenetic structures than lineages of different ecological background or age.

## 2 MATERIALS AND METHODS

### 2.1 Study area, sampling, and environmental variables

We use data from 40 transects (5 m × 500 m) that were inventoried for several plant groups along a 500‐km stretch of the Juruá river in western Brazilian Amazonia as described by Tuomisto et al. (2016) (Figure 1). In each transect, the plants were counted and identified to species by experts. Here we use the data on ferns and palms.

The transects were distributed along the Juruá river so as to cover the pre‐identified landscape heterogeneity, especially the proposed geological boundary between the Solimões formation and the Içá formation (Higgins et al., 2011; Schobbenhaus et al., 2004). Nine transects were located on the Solimões formation, 12 on the Içá formation, and 19 on old river terraces, which are flat relatively low‐lying areas that are currently non‐inundated. Each transect is considered as an independent unit in the analyses. We focus on the sum of exchangeable base cations (Ca, K, Mg, and Na as expressed in cmol(+)/kg), because it has been found to be highly correlated with the strongest floristic gradient both in the Juruá area (Tuomisto et al., 2016) and elsewhere in Amazonia (Higgins et al., 2011; Phillips et al., 2003; Pitman et al., 2008; Tuomisto et al., 2003, 2019). Base cation concentration has also been used in previous community phylogenetic analyses of ferns (Lehtonen et al., 2015) and palms (Muscarella et al., 2019) in Amazonia.

Among the transects analyzed here, base cation concentration means and ranges were 0.16 (0.11–0.26) cmol(+) kg^−1^ on the Içá formation, 0.37 (0.18–0.60) cmol(+) kg^−1^ on fluvial terraces and 7.59 (2.56–13.89) cmol(+) kg^−1^ on the Solimões formation. For details on the field sampling and soil sample analyses see Tuomisto et al. (2016) and Balslev et al. (2019). The entire study area is well within the lowland Amazonian rain forest area, and we expect all transects to share the same climate and regional species pool.

### 2.2 Phylogenies

For quantifying the palm community structure, we randomly resampled 100 trees from the posterior distribution of the global palm phylogeny of Faurby et al. (2016) and pruned these trees to only keep the taxa observed in our transects.

To analyze the phylogenetic structure of fern communities, we compiled a data matrix of three plastid markers (*atpB*, *rbcL*, *rps4*) representing the observed fern taxa. Sequence data were either downloaded from Genbank or newly produced for this study (see Appendix 1) following standard laboratory procedures and using primers ATPB672F (Wolf, 1997) and ATPE384R (Pryer et al., 2004) for *atpB*, ESRBCL1F and ESRBCL1361R (Korall et al., 2006), or in the case of *Danaea* aF (Hasebe et al., 1994) and F1379R (Wolf et al., 1999) for *rbcL*, and rps4.5’ (Small et al., 2005) and trnS^GGA^ (Shaw et al., 2005) for *rps4*. The ferns *Nephrolepis biserrata* and *N. rivularis* were both present in the study area but were treated as a single taxon in the analyses due to difficulties in field identification. Altogether, the final matrix was composed of 140 terminal taxa and 2833 bp of sequence data aligned with default parameters in Mafft 7.215 (Katoh & Standley, 2013).

A parsimony analysis was run in TNT 1.5 (Goloboff et al., 2008) to produce a starting tree for the subsequent molecular dating analysis. We used nine fossil calibration points (Table 1) to date the fern phylogeny in Beast 1.8.4 (Drummond et al., 2012) by running seven independent chains, each consisting of 1 × 10^8^ generations and sampling every 1000 generations. Data were partitioned by genes and a GTR +G model with four rate categories was assigned to each partition. Site and clock models were unlinked between the partitions. After verifying the convergence of the runs (effective sample sizes exceeded 1000 for the sampled parameters) in Tracer (Rambaut et al., 2018), a random set of 100 trees were sampled from the posterior for downstream community structure analyses. The aligned sequence data and a maximum clade credibility tree from the full posterior sample are available at TreeBase (S28880).

**TABLE 1:** Calibration points for the ages in the fern phylogeny. Each calibration was assigned to the stem lineage. Justifications for the soft maximum ages (95% quantiles) follow Lehtonen et al. (2017)

Clade	Hard min age	Soft max age	Reference
Marattiaceae	318 Ma	407 Ma	Stewart and Rothwell (1993)
Hymenophyllaceae	228 Ma	318 Ma	Axsmith et al. (2001)
Cyatheaceae	125 Ma	251 Ma	Smith et al. (2003)
Schizaeaceae	112 Ma	251 Ma	Skog (1993)
Lindsaeaceae	99.6 Ma	201.6 Ma	Schneider and Kenrick (2001)
Dennstaedtiaceae	70.6 Ma	201.6 Ma	Serbet and Rothwell (2003)
Blechnaceae	55.8 Ma	145.5 Ma	Wang et al. (2006)
*Pleopeltis*	15.8 Ma	91.5 Ma	Schneider et al. (2015)
*Elaphoglossum*	15 Ma	81.1 Ma	Lóriga et al. (2014)

### 2.3 Phylogenetic community structure analyses

**FIGURE 2:**
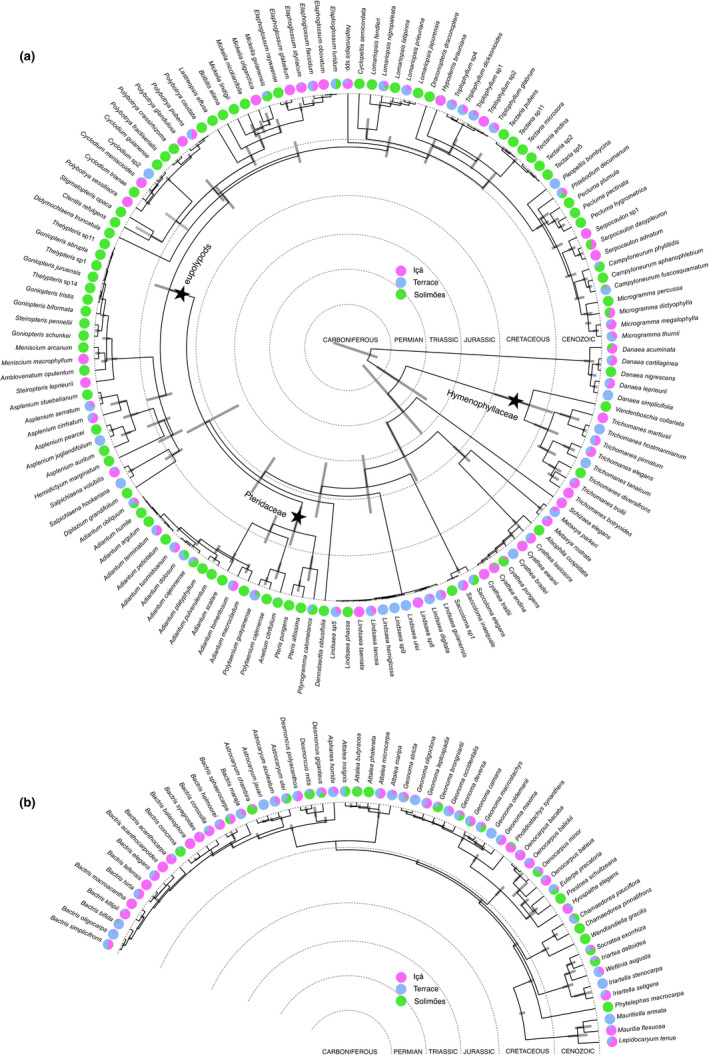
Fern (a) and palm (b) phylogenies with species' relative densities on the three geological formations mapped on the tips. The three fern subclades that were also analyzed separately are marked with stars

We treated each transect as a local community and computed for each of them a set of different phylogenetic community metrics using presence‐absence data. In order to take different evolutionary ages of ferns and palms into account when comparing phylogenetic community patterns, we separately analyzed three fern clades of roughly the same crown‐group age as palms (~100 Ma): Hymenophyllaceae, Pteridaceae, and eupolypods (Figure 2a). The phylogenetic community metrics were calculated in R v.3.6.2 (R Core Team, 2019) with packages ‘ape’ (Paradis et al., 2004), ‘betapart’ (Baselga & Orme, 2012), ‘picante’ (Kembel et al., 2010), and ‘vegan’ (Oksanen et al., 2010) over 100 trees that were randomly sampled from the posterior.

Compositional dissimilarity between two communities (turnover, often referred to as β‐diversity) was measured as the proportion of taxa not shared between them (complement of the classical Sørensen index based on presence‐absence data) and phylogenetic dissimilarity as the proportion of phylogenetic branch lengths not shared (PhyloSørensen, PhyloSor). These were illustrated with ordination diagrams based on Principal Coordinates Analysis (PCoA). A standardized version of PhyloSørensen was also obtained by relating the observed values to a null distribution drawn from 1000 randomizations (each of the 100 trees randomized ten times) by shuffling tips of the phylogenies (SES.PhyloSor). The other studied metrics were: S (species richness), SES.PD (standard effect size of Faith's phylogenetic diversity; Faith, 1992), SES.MPD (standard effect size of mean phylogenetic pairwise distance; Webb et al., 2002), SES.MNTD (standard effect size of mean nearest taxon distance; Webb, 2000), and VPD (Λ+, variance of pairwise distances, Clarke & Warwick, 2001). We did not standardize the VPD as it is insensitive to sampling effort, thus justifying direct comparison of observed values despite variable species richness (Clarke & Warwick, 2001). For standardizing the other metrics, we applied an independent swap algorithm (Gotelli & Entsminger, 2003) for the null model using as the species pool all the observed species belonging to the relevant taxon (ferns, palms, Hymenophyllaceae, Pteridaceae, eupolypods). For the null model, we generated 999 random communities separately for each of 100 phylogenies and used the median value of the studied metric across the phylogenies for further analyses. We set the include.root argument to FALSE when computing SES.PD to avoid biased estimations in ‘picante’ (Molina‐Venegas, 2019).

S and SES.PD quantify the amount of evolutionary history present in a community, whereas SES.MPD and SES.MNTD describe how phylogenetically divergent the communities are, and VPD reflects the unevenness of the community phylogenetic tree (Tucker et al., 2017). Typically, S and SES.PD are strongly correlated, but they may deviate, for example, between a community composed of a recent in‐situ radiation and a community assembled from distantly related species (Faith, 1992). Both SES.MPD and SES.MNTD average the phylogenetic distances within a community, with SES.MPD being more strongly affected by the deep phylogenetic divergences, and SES.MNTD emphasizing the phylogenetic proximity of the closest relatives in the community (Tucker et al., 2017). VPD responds to uneven structure of the community phylogenetic tree: it will be elevated when a community is composed of both relatively closely and distantly related members, in contrast to a community composed of members with more equal phylogenetic distances (Clarke & Warwick, 2001). The standardized metrics describe how the observed communities deviate from a random expectation, with negative values indicating closer phylogenetic relationships than expected (phylogenetic clustering) and positive values greater than expected lineage divergence (phylogenetic overdispersion), respectively. Linear and second‐order polynomial regression analyses were run to test whether the studied community metrics were significantly related to soil base cation concentration.

## 3 RESULTS

The 40 transects contained a total of 140 fern species and 60 palm species. The Hymenophyllaceae clade had nine, Pteridaceae 19, and eupolypods 82 species. Of all the fern species, 39% were found only on the Solimões formation (soils with high soil base cation concentration), 9% only on the Içá formation (soils with low base cation concentration), and 7% only on the terraces (soils with low‐intermediate base cation concentration). For palms, the percentages of species recorded exclusively in these same formations were 7%, 5%, and 7%, respectively. Thus, altogether 56% of the fern species, but only 18% of the palm species, were found exclusively on a single terrain type. The estimated crown‐group ages were as follows: ferns 330 Ma (318–385 Ma), palms 106 Ma (100–109 Ma), Hymenophyllaceae 92 Ma (56–144 Ma), Pteridaceae 90 Ma (69–127 Ma), and eupolypods 95 Ma (73–115 Ma) (Figure 2).

**FIGURE 3:**
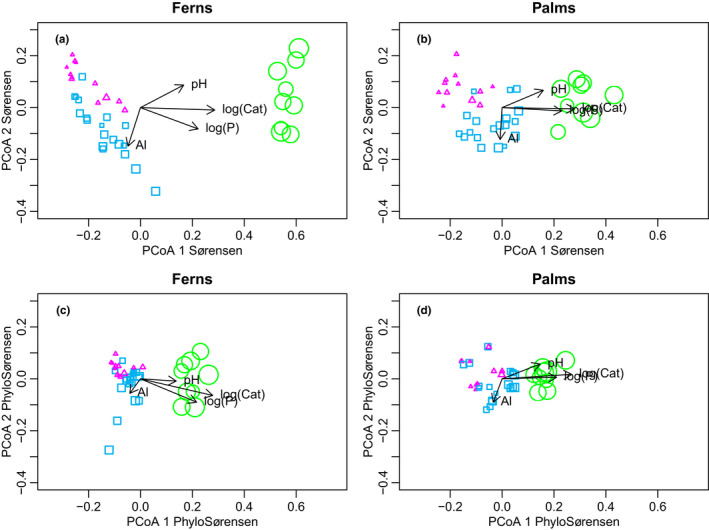
Ordinations (Principal Coordinates Analysis) of 40 inventory transects based on species composition of ferns (a,c) and palms (b,d). Compositional dissimilarities were based on presence‐absence data using either the classical Sørensen index (a,b) or the PhyloSørensen (c,d) index. The latter takes into account the length of the shared evolutionary history as quantified in the relevant phylogeny in Figure 2. Symbol sizes are proportional to the concentration of exchangeable base cations in the soil (Ca + Mg + K + Na) and symbols are colored according to the geological formation (Içá pink triangles, Solimões green circles, terrace blue squares). Arrows indicate correlations of the first two PCoA axes with chemical properties of the soils: logarithmically transformed base cation concentration, log‐transformed phosphorus concentration, aluminum concentration, and pH

Ordinations based on the Sørensen index divided the communities into two clear groups, with all Solimões formation transects in one group and all the Içá formation and terrace transects in the second group. The first PCoA axis most strongly correlated with the soil cation concentration (Figure 3). The contrast was clearer for ferns than for palms, but the basic pattern was similar for both. The terrace transects and Içá formation transects also clustered into their own groups, but these were not very clearly separated from each other (Figure 3a,b). When phylogenetic relationships were taken into account (PhyloSor), all groupings became less distinct and the Içá and terrace transects got more mixed (Figure 3c,d).

**FIGURE 4:**
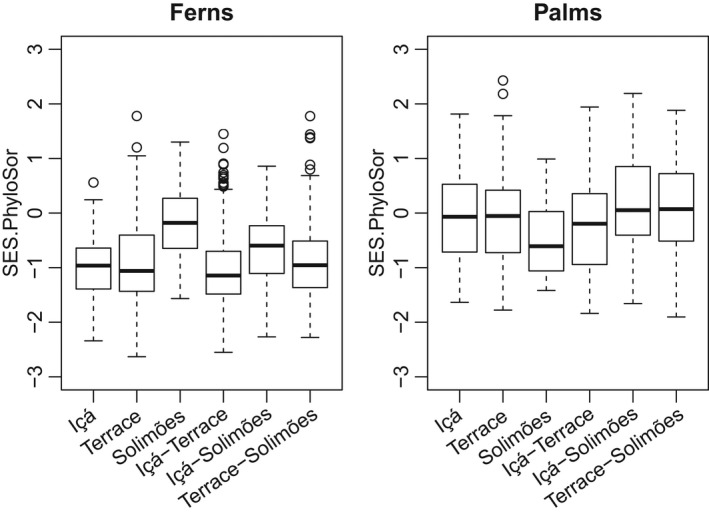
Boxplots of the values of SES.PhyloSørensen both for transects on the same geological surface type and for transects on different geological surface types. Values close to zero indicate that phylogenetic distances of communities on or between geological surface types do not deviate from random expectation, whereas values below zero indicate that observed phylogenetic distances are smaller than expected by chance

Fern communities on the Solimões formation were, on average, as phylogenetically distinct as would be expected by chance (SES.PhyloSor close to zero; Figure 4). On and across the other geological formations, phylogenetic distances among transects were smaller than expected by chance. For palms, the situation was the reverse: in most comparisons, phylogenetic distances were as expected by chance, but between transects on the Solimões formation, phylogenetic distances were smaller than expected.

**FIGURE 5:**
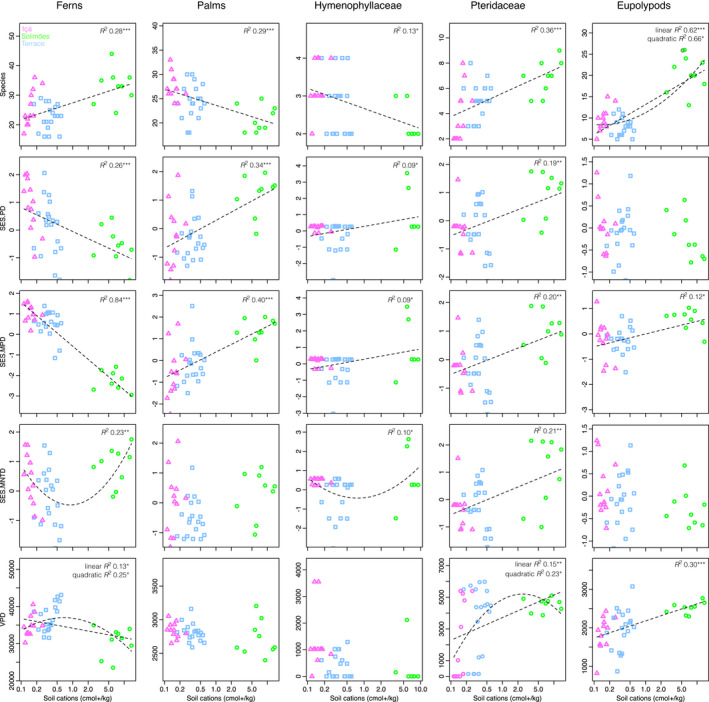
Species richness, standard effect size of phylogenetic diversity (SES.PD), standard effect size of mean pairwise phylogenetic distance (SES.MPD), standard effect size of mean nearest taxon distance (SES.MNTD), and variance of pairwise distances (VPD) of fern and palm communities along the Juruá river. The indices were also computed separately for Hymenophyllaceae, Pteridaceae, and eupolypod ferns, that is, three fern clades that have roughly equal crown‐group age (~100 Ma) as palms. Linear and second‐order polynomial regression lines with corresponding explanatory power (adjusted *R* ^2^) are shown if statistically significant (*p* < .05)

The studied lineages showed distinct patterns in their phylogenetic community structure (Figure 5). In the fern communities, species richness increased significantly with increasing soil base cation concentration, whereas VPD, SES.PD, and SES.MPD decreased. SES.MNTD had a U‐shaped pattern with positive values toward both ends of the gradient. The patterns observed in palm communities contrasted those in ferns. Palm species richness decreased with increasing soil base cation concentration, whereas SES.PD and SES.MPD increased. For palms, no patterns were detected in VPD or SES.MNTD.

Each fern subclade responded differently to the soil gradient. With increasing soil base cation concentration, eupolypods showed a significant increase in species richness, VPD, and SES.MPD, and no signal in SES.PD or SES.MNTD. In Pteridaceae, all the investigated community metrics increased significantly with increasing soil cation concentration. Hymenophyllaceae showed a pattern that was similar to that in palms, with decreasing species richness and increasing SES.PD, SES.MPD, and SES.MNTD (the last one not significant in palms) with increasing soil base cation concentration.

## 4 DISCUSSION

Edaphic variation is known to control floristic composition within climatically determined vegetation types, but the underlying mechanisms by which this control takes place are not fully understood (Hulshof & Spasojevic, 2020). The phylogenetic structure of communities along edaphic gradient may help to illuminate these mechanisms. As a first step, it is crucial to understand on what scale the phylogenetic community structure varies. Previous studies on the phylogenetic structure of Amazonian plant communities have generally not distinguished between different geological units within the *terra firme* forests, except for the most extreme white sands and occasionally terraces (Fine & Kembel, 2011; Honorio Coronado et al., 2015; Umaña et al., 2012). However, phylogenetic variation in *terra firme* community structure has been noticed, even if not specifically investigated, before (Fine & Kembel, 2011; Schreeg et al., 2010). Here we have demonstrated that the Solimões‐Içá boundary within *terra firme* is associated not only with a floristic turnover, but with significantly different phylogenetic structures of fern and palm communities across the boundary. The phylogenetic community structure thus significantly varies over much shorted edaphic gradient in Amazonia than commonly thought. This indicates that edaphic conditions maintain communities with unique evolutionary histories.

We found that when phylogeny was considered, floristic dissimilarities between the communities on different soils were somewhat less clear than when phylogeny was not considered (Figure 3). This can be explained if adaptive radiation and associated niche shifts play some role in the evolutionary history of the communities, as then niche shifts sometimes cause phylogenetically close species to become ecologically distinct. In such a case, the simple species‐level dissimilarity reflects the environmental relationships of a local community more accurately than phylogenetically informed dissimilarity does. This is an example of how combining phylogenetic β‐diversity with traditional measures of species turnover (or β‐diversity) can lead to better insights into the mechanism underlying diversity patterns (Graham & Fine, 2008) revealing, in this case, that edaphic niches are not entirely phylogenetically conserved.

We furthermore tested if phylogenetic β‐diversity between communities deviated from a random expectation. It turned out that, on average, local fern communities on the cation‐rich soils of the Solimões formation were as phylogenetically distinct as would be expected by chance and that the same was true for the palm communities that were on the cation‐poor soils of the other formations (Figure 4). In other words, these communities represent phylogenetically rather random samples of the available species pool. This is understandable, given the widespread preference for cation‐rich soils among ferns and for cation‐poor soils among palms. In contrast, the poor‐soil fern communities and the rich‐soil palm communities only sample a part of the phylogenetic diversity available in the species pool, as shown by their smaller than expected phylogenetic β‐diversity.

We have earlier suggested that niche shifts in ferns may happen more often from poor to rich soils than vice versa (Lehtonen et al., 2015; Tuomisto et al., 2014). Although this hypothesis should preferably be tested by investigating the species’ soil preferences on a complete phylogeny, biased niche shifts should leave a trace in the phylogenetic structure of communities. If cation‐poor soils indeed are more difficult to adapt to, then the poor‐soil specialists should be concentrated into those few lineages that have acquired the capacity to thrive on and speciate on poor soils, which would be reflected in a high VPD in poor‐soil fern communities. In contrast, on more cation‐rich soils the fern communities should be phylogenetically more evenly assembled and have a relatively low VPD, due to more common niche shifts toward cation‐rich soils across the fern phylogeny. Our results conformed with the predicted pattern for ferns (Figure 5). This result is in line with the random‐like phylogenetic β‐diversity observed in the rich‐soil fern communities, which is likewise consistent with these communities being a more representative sample of the phylogeny than the poor‐soil fern communities are.

Easier adaptation to more nutrient‐rich soils has also been suggested to be a driver of phylogenetic community structure of Amazonian trees (Honorio Coronado et al., 2015). The presumed bias in niche shifts toward cation‐rich soils could explain why Amazonian fern diversity is higher on cation‐rich soils despite the apparent long‐term prevalence and the possible ancestral preference of Amazonian plants for poor soils (Frasier et al., 2008; Kubitzki, 1989).

However, these interpretations only apply when ferns are considered as one group. When the three subclades of ferns were analyzed separately, they showed either no relationship or an increase in VPD with increasing soil base cation concentration. This may be related to differences in the relevant temporal scale, in ancestral ecologies, or in lineage‐specific phylogenetic constraints. Both Pteridaceae and Eupolypods showed an increase in VPD along the soil cation concentration gradient, and both are predominantly composed of species with a preference for rich soils (Figure 2a), and this is probably their ancestral state.

Our results confirmed the earlier observation that the phylogenetic structure of Amazonian palm communities contrasts that of fern communities (Muscarella et al., 2019). Fern communities had highest species richness, but lowest phylogenetic diversity (SES.PD and SES.MPD) on rich soils, and palms had exactly the opposite pattern (Figure 5). These patterns, together with the U‐shaped response of SES.MNTD in fern communities to the soil cation gradient, reveal that the phylogenetic community patterns in ferns are driven by strong preference of the deepest fern lineages for cation‐poor soils (Figure 2a). It is noteworthy that cation‐poor soils likely represent the ancestral habitat for Amazonian palms as well (Figure 2b), yet their phylogenetic community structure is very different and species richness of palms remains higher on relatively poor soils. Ecologically, palms differ from ferns by their presumably more restricted dispersal capacity, stronger biotic interactions with pollinators and seed dispersers, and apparently broader edaphic niches. In the study area, more than half of the fern species were found only on a single geological formation, but most of the palm species on at least two formations (Figure 2; Tuomisto et al., 2016). Therefore, the geological boundary appears to be a less intense filter for palms than it is for ferns, as can also be seen from the ordinations based on species turnover (Figure 3).

Compositional dissimilarity between communities can result from various factors, like competition, dispersal limitation, habitat filtering, and speciation (Franklin et al., 2013). The strength of competition between vascular plants does not seem to depend on their phylogenetic relatedness (Cahill et al., 2008), so it seems unlikely that the observed soil‐related phylogenetic patterns would have emerged from competition. Random ecological drift of ecologically equivalent species is also an unlikely explanation for such a clear relationship between soils and community phylogenetic patterns. Dispersal limitation is not a plausible explanation for the disjunct phylogenetic structure across the Solimões‐Içá boundary either: if it were, ferns (with efficient wind dispersal of tiny spores) should show weaker rather than stronger edaphic signal than palms (with heavy animal‐dispersed seeds). Hence, habitat filtering appears the most likely explanation for the observed pattern. Environmental filtering is expected to result in phylogenetic clustering if niches are conserved. The cation‐poor soils can be expected to be a harder environment and therefore more strongly filtered and phylogenetically clustered. In ferns, we observed the opposite pattern, although all the fern subclades as well as palms had more clustered communities on poor soils. As explained above, the pattern in ferns is likely caused by the phylogenetically biased preference for poor soils in deepest lineages of the strongly imbalanced and very deep phylogeny. This makes the SES.MPD, which gives most weight on the deepest lineages, to show opposite pattern in ferns compared to palms and fern subclades. It is worth noting that in ferns the SES.MNTD, which emphasizes the patterns toward the tips of the tree, is contrasting the SES.MPD. We therefore conclude that the contrasting phylogenetic patterns between fern and palm communities are caused by the vastly different phylogenetic depth of the communities under comparison and by the poor‐soil preference of the deepest fern lineages but prevalence of rich‐soil preference in the younger lineages.

Our analyses using three separate fern subclades confirmed that different fern lineages show different trajectories. Each of the fern subclades was estimated to have originated in the mid‐Cretaceous, so their ages correspond to that of palms. All fern subclades responded significantly, yet differently, to the soil fertility gradient (Figure 5). Distinct clade‐specific responses to soil variables were observed in rainforest tree communities already a long time ago (Gentry, 1988). These patterns illustrate that lineages have their own evolutionary trajectories and that more inclusive clades can be expected to include more heterogeneous phylogenetic patterns.

Out of the three fern subclades, the Hymenophyllaceae communities resembled palm communities in their phylogenetic structure. Both palms and Hymenophyllaceae had higher species richness on poor soils. The similarity in the phylogenetic community structure between these two groups suggests that under common environmental conditions, even very distantly related lineages may converge to similar phylogenetic community structure over time. This conclusion will need to be tested with other plant groups, as the small number of Hymenophyllaceae species per community seriously limits the robustness of our interpretation.

Edaphic factors have been suggested to be the key driver of speciation in the Mediterranean plant diversity hot spot (Buira et al., 2021). Floristic heterogeneity and its link with soil properties in the highly diverse Amazonian forests has also been known for quite some time (Gentry, 1988; Tuomisto et al., 1995; Young & León, 1989). It now appears that the edaphically determined floristic variation reflects independent phylogenetic histories of these floras and that abrupt edaphic gradients, such as the Solimões‐Içá boundary studied here, maintain the evolutionary independence by environmental filtering and enhancing in situ speciation. In an Amazonian context, this may result in each geo‐ecologically defined landscape unit having its own unique evolutionary regime, which could result in even higher basin‐wide diversity than the number of different niches alone would predict. These patterns may have gone unnoticed in part because it has been common to treat all the *terra firme* communities as a single unit, or the phylogenetic responses of different lineages may have become obscured when the focus of analysis has been on phylogeny‐wide signals within taxonomically broad communities that are not defined phylogenetically but, for example, structurally (e.g., canopy trees). Future studies on Amazonian diversity and community ecology would greatly benefit from incorporating geological history into combined analyses of ancestral ranges and habitat preferences (Tuomisto, 2007; Wiens, 2012).

## CONFLICT OF INTEREST

The authors have no competing interests to declare.

## AUTHOR CONTRIBUTIONS

**Samuli Lehtonen:** Conceptualization (equal); Formal analysis (lead); Writing – original draft (lead). **Robert Muscarella:** Conceptualization (supporting); Formal analysis (supporting); Writing – review & editing (supporting). **Gabriel Massaine Moulatlet:** Data curation (equal); Writing – review & editing (supporting). **Henrik Balslev:** Data curation (equal); Funding acquisition (equal); Writing – review & editing (supporting). **Hanna Tuomisto:** Conceptualization (equal); Data curation (lead); Formal analysis (supporting); Funding acquisition (equal); Writing – review & editing (equal).

## Data Availability

All the data can be openly accessed. DNA sequences are available at GenBank (see Appendix 1 for accession codes). The environmental data and palm data were published in Balslev et al. (2019). The palm phylogeny was published in Faurby et al. (2016). The fern phylogeny is available at TreeBASE (S25312) and the other fern data can be accessed at Zenodo https://doi.org/10.5281/zenodo.5602933.

## APPENDIX 1

Taxonomic sampling for the fern community phylogenies with GenBank accession numbers for *rbcL*, *atpB*, and *rps4*. Sequences newly obtained in this study written in bold. A dash (–) indicates not available.

*Adiantum argutum* Splitg. KJ628525, KJ716378, MN781356; *Adiantum cajennense* Willd. ex Klotzsch KJ628527, MN781256, MN781364; *Adiantum decoratum* Maxon & Weath. MN781316, MN781242, MN781366; *Adiantum diogoanum* Glaziou, KJ628557, MN781263, –; *Adiantum dolosum* Kunze, KJ628558, –, MN781365; *Adiantum humile* Kunze, KJ628566, –, MN781360; *Adiantum lucidum* (Cav.) Sw., KJ628575, –, –; *Adiantum macrocladum* Klotzsch, KJ628577, MN781266, MN781357; *Adiantum obliquum* Willd., KJ628581, MN781258, MN781362; *Adiantum paraense* Hieron., KJ628584, MN781257, MN781363; *Adiantum petiolatum* Desv., KJ628586, MN781265, MN781358; *Adiantum platyphyllum* Sw., KJ628590, MN781261, –; *Adiantum poeppigianum* C. Presl., MN781347, MN781285, MN781367; *Adiantum pulverulentum* L., KJ628544, MN781262, –; *Adiantum scalare* R.M. Tryon, KJ628594, MN781259, –; *Adiantum* sp6, MN781346, MN781287, MN781368; *Adiantum* sp8, MN781345, MN781288, MN781369; *Adiantum terminatum* Kunze, KJ628599, MN781264, MN781359; *Adiantum tomentosum* Klotzsch, KJ628601, MN781255, MN781355; *Adiantum tuomistoanum* J. Prado, KJ628609, MN781260, MN781361; *Alsophila cuspidata* (Kunze) D.S. Conant, KR082855, AM176584, AM176479; *Amblovenatum opulentum* (Kaulf.) J.P. Roux, EF463295, EF463551,–; *Ananthacorus angustifolius* (Sw.) Underw. & Maxon, KX164951, –, –; *Anetium citrifolium* (L.) Splitg., KU517578, EF452017, –; *Asplenium auritum* Sw., KF992426, EF463327, AY549759; *Asplenium cirrhatum* Rich. ex Willd., KJ628626, MN781250,–; *Asplenium delitescens* (Maxon) L.D.Gómez, KJ628629, –, –; *Asplenium hallii* Hook., MN781344, –, –; *Asplenium juglandifolium* Hook., EF463151, EF463333, AY459168; *Asplenium pearcei* Baker, KJ628630, KM114064, –; *Asplenium serratum* L., KJ628636, MN781249,–; *Asplenium stuebelianum* Hieron., KJ628638, MN781251, –; *Bolbitis aliena* Alston, KJ628643, –, GU376646; *Campyloneurum angustifolium* Fée, AF470344, AY459515, AY362645; *Campyloneurum aphanophlebium* T. Moore, KJ628649, MN781243, EU250352; *Campyloneurum fuscosquamatum* Lellinger, KJ628654, –, –; *Campyloneurum phyllitidis* C. Presl, KJ628658, MN781244, EU250354; *Ctenitis refulgens* (Mett.) Vareschi, KY701654, KM114092, –; *Cyathea andina* Domin, KT235814, –, –; *Cyathea bradei* (P.G. Windisch) Lellinger, KJ628672, KM114102, KP244090; *Cyathea ewanii* Alston, MN781342, MN781289, MN781350; *Cyathea lasiosora* Domin, KJ628675, KJ716375, –; *Cyathea leucolepismata* Alston, MN781341, –, MN781351; *Cyathea pungens* Domin, KR082853, –, –; *Cyathea traillii* Domin, –, –, MN781352; *Cyclodium guianense* (Klotzsch) L.D.Gómez, KT272937, KM114083, KT273010; *Cyclodium meniscioides* (Willd.) C. Presl, KT272939, MN781240, KT273012; *Cyclodium* sp2, MN781348, MN781241, –; *Cyclodium trianae* (Mett.) A.R. Sm., KJ628681, EF463386, KU521898; *Cyclopeltis semicordata* (Sw.) J. Sm., EF463234, EF463480, KY397977; *Danaea acuminata* Tuomisto & R.C. Moran, EU221744, EU221683, –; *Danaea cartilaginea* Christenh. & Tuomisto, EU221753, EU221691, –; *Danaea leprieurii* Kunze, EU439077, EU439055, EU439097; *Danaea nigrescens* Jenman, KJ628690, KJ628511, –; *Danaea simplicifolia* Rudge, EU221791, EU221726, –; *Dennstaedtia obtusifolia* (Willd.) T. Moore, –, –, MN781370; *Dicranoglossum desvauxii* (Klotzsch) Proctor, AY362584, –, AY362657; *Didymochlaena truncatula* (Sw.) J. Sm., DQ508769, EF452030, AF425161; *Diplazium grandifolium* Sw., KJ628700, –, –; *Diplazium* sp6, –, –, MN781371; *Diplazium tabalosense* Hieron., MN781340, MN781308, MN781372; *Draconopteris draconoptera* (D.C. Eaton) Li Bing Zhang & Liang Zhang, KJ628848, MN781245, KU605088; *Elaphoglossum flaccidum* (Fée) T. Moore, EF463192, EF463405, AY540246; *Elaphoglossum glabellum* J. Sm., AY818691, KM114081, AY536167; *Elaphoglossum luridum* Christ., AY534859, MN781274, KJ528164; *Elaphoglossum obovatum* Mickel, KJ628708, –, –; *Elaphoglossum raywaense* (Jenman) Alston, –, –, MN781373; *Elaphoglossum styriacum* Mickel, MN781339, MN781290, MN781374; *Goniopteris abrupta* (Desv.) A.R. Sm., KJ628858, KM114084, –; *Goniopteris biformata* (Rosenst.) Salino & T.E. Almeida, MN781327, –, MN781388; *Goniopteris juruensis* (C. Chr.) Brade, –, –, MN781390; *Goniopteris lugubriformis* (Rosenst.) Salino & T.E. Almeida, MN781325, –, MN781393; *Goniopteris schunkei* (A.R. Sm.) Salino & T.E. Almeida, –, MN781281, MN781395; *Goniopteris tristis* (Kunze) Brade, MN781321, MN781282, MN781399; *Hemidictyum marginatum* (L.) C. Presl, EF463318, KT329387, KT329410; *Hypoderris brauniana* (H. Karst.) F.G. Wang & Christenh., KJ628847, –, –; *Lastreopsis effusa* (Sw.) Tindale, KJ464441, EF463422, KJ628939; *Lindsaea digitata* Lehtonen & Tuomisto, KJ628723, MN781276, GU478698; *Lindsaea falcata* Dryand., KJ628728, MN781277, GU478702; *Lindsaea guianensis* (Aubl.) Dryand., KJ628729, MN781267, GU478700; *Lindsaea hemiglossa* K.U. Kramer, KJ628731, MN781268, GU478660; *Lindsaea lancea* (L.) Bedd., KJ628733, MN781269, KJ628959; *Lindsaea phassa* K.U. Kramer, MN781311, –, GU478659; *Lindsaea* sp5, MN781338, MN781291, MN781375; *Lindsaea* sp8, MN781312, –, MN781353; *Lindsaea* sp9, MN781313, –, MN781354; *Lindsaea taeniata* K.U. Kramer, KJ628753, MN781272, GU478655; *Lindsaea ulei* Hieron, KJ628756, MN781273, KJ628956; *Lomariopsis fendleri* D.C. Eaton, MN781336, MN781292, MN781376; *Lomariopsis japurensis* (C. Martius) J. Sm., KJ628760, MN781306, KJ628952; *Lomariopsis latipinna* Stolze, MN781337, MN781293, MN781377; *Lomariopsis nigropaleata* Holttum, MN781334, MN781294, MN781378; *Lomariopsis prieuriana* Fée, KJ628765, KJ716387, –; *Meniscium arcanum* (Maxon & C.V. Morton) Pic. Serm., MN781328, MN781279, MN781387; *Meniscium lingulatum* (C. Chr.) Pic. Serm., –, MN781283, MN781392; *Meniscium macrophyllum* Kunze, KJ628869, KM114066, KT805106; *Metaxya parkeri* (Hook. & Grev.) J. Sm., KP244086, –, KP244094; *Metaxya rostrata* (Kunth) C. Presl, KP244085, MN781278, KP244107; *Mickelia guianensis* (Aubl.) R.C. Moran, Labiak & Sundue, EF463208, EF463426, GU376698; *Mickelia lindigii* (Mett.) R.C. Moran, Labiak & Sundue, MN781343, –, MN781349; *Mickelia nicotianifolia* (Sw.) R.C. Moran, Labiak & Sundue, EF463171, EF463382, GU376666; *Mickelia oligarchica* (Baker) R.C. Moran, Labiak & Sundue, KJ464489, –, GU376667; *Microgramma baldwinii* Brade, KY847861, KM114086, KY847866; *Microgramma dictyophylla* (Kunze ex Mett.) de la Sota, MN781335, MN781295, MN781379; *Microgramma megalophylla* (Desv.) de la Sota, AY362578, –, AY362650; *Microgramma percussa* (Cav.) de la Sota, MN781332, MN781296, MN781380; *Microgramma thurnii* (Baker) R.M. Tryon & Stolze, KJ628786, –, –; *Nephrolepis biserrata* (Sw.) Schott, KJ628787, KM114089, HM748170; *Nephrolepis rivularis* (Vahl.) Mett. ex Krug, HM748158, –, HM748184; *Pecluma hygrometrica* (Splitg.) M.G. Price, KT780736, –, KT794113; *Pecluma pectinata* (L.) M.G. Price, KT780741, –, KT794120; *Pecluma plumula* (Willd.) M.G. Price, KT780745, –, KT794124; *Phlebodium decumanum* J. Sm., MN781333, MN781300, MN781381; *Pityrogramma calomelanos* (L.) Link, KJ628794, KM007687, KM007800; *Pleopeltis bombycina* (Maxon) A.R. Sm., EU650136, DQ642213, –; *Polybotrya caudata* Kunze, AB232393, KM114099, –; *Polybotrya crassirhizoma* Lellinger, KT272958, MN781307, KT273035; *Polybotrya fractiserialis* (Baker) J. Sm., KM114201, KM114098, –; *Polybotrya glandulosa* Mett. ex Kuhn, KJ628799, MN781238, –.; *Polybotrya pubens* Kunze, KT272983, MN781310, KT273063; *Polybotrya sessilisora* R.C. Moran, KJ628803, MN781239, KT273069; *Polytaenium cajenense* (Desv.) Benedict, KX165027, EF452052, –; *Polytaenium guayanense* (Hieron.) Alston, KX165033, MN781248, –; *Pteris altissima* Poir., KM008147, KM007698, KM007813; *Pteris pungens* Willd., KM008215, KM007763, KM007880; *Saccoloma elegans* Kaulf., KJ628821, HQ157279, GU478639; *Saccoloma inaequale* (Kunze) Mett., KJ628822, EU352283, KJ628955; *Saccoloma* sp1, MN781331, MN781304, MN781382; *Salpichlaena hookeriana* Alston, KJ628824, MN781254, KJ628937; *Salpichlaena volubilis* (Kaulf.) J. Sm., KJ628827, EF463357, KU898601; *Schizaea elegans* (Vahl) Sw., NC_035807, NC_035807, NC_035807; *Serpocaulon adnatum* (Kunze ex Klotzsch) A.R. Sm., AY362593, –, AY362666; *Serpocaulon dasypleuron* (Kunze) A.R. Sm., –, –, EF551089; *Serpocaulon* sp1, MN781330, MN781299, MN781383; *Steiropteris leprieurii* (Hook.) Pic. Serm. var. *incana* (Christ) A.R. Sm., –, MN781286, MN781391; *Steiropteris leprieurii* (Hook.) Pic. Serm. var. *leprieurii*, MN781326, –, MN781389; *Steiropteris pennellii* (A.R. Sm.) Salino & T.E. Almeida, MN781324, MN781284, MN781394; *Stigmatopteris opaca* C. Chr., KU521891, –, –; *Tectaria andina* C. Chr., MN781314, –, –; *Tectaria microsora* A.R. Sm., –, –, MN781384; *Tectaria pilosa* (Fée) R.C. Moran, KJ628852, –, –; *Tectaria pubens* R.C. Moran, KF887193, –, –; *Tectaria* sp11, –, –, MN781385; *Tectaria* sp12, MN781329, MN781302, MN781386; *Tectaria* sp2, MN781315, –, –; *Tectaria* sp5, MN816393, –, –; *Thelypteris* sp1, MN781323, –, MN781398; *Thelypteris* sp11, –, MN781297, MN781396; *Thelypteris* sp14, MN781322, MN781298, MN781397; *Trichomanes botryoides* Kaulf., KJ628885, MN781246, –; *Trichomanes diversifrons* (Bory) Mett. ex Sadeb., KJ628889, MN781271, –; *Trichomanes elegans*Rich., KP153244, MN781247, EU553534; *Trichomanes hostmannianum* (Klotzsch) Kunze, AB257500, KJ716385, –; *Trichomanes martiusii* C. Presl, KJ628894, MN781270, –; *Trichomanes pinnatum* Hedw., KJ628904, EF463474, –; *Trichomanes* sp1, MN781317, MN781275, –; *Trichomanes tanaicum* J.V. Sturm, –, MN781280, MN781400; *Trichomanes trollii* Bergdolt, MH348274, MH348274, MH348274; *Triplophyllum dicksonioides* (Fée) Holttum, KJ628916, MN781253, KF667571; *Triplophyllum funestum* (Kunze) Holttum, KJ628920, EF463532, KF667573; *Triplophyllum glabrum* J. Prado & R.C. Moran, KF887207, MN781252, –; *Triplophyllum* sp1, –, MN781303, MN781401; *Triplophyllum* sp2, MN781320, MN781305, MN781402; *Triplophyllum* sp3, MN781318, MN781301, MN781404; *Triplophyllum* sp4, MN781319, MN781309, MN781403; *Vandenboschia collariata* (Bosch) Ebihara & K. Iwats., FJ460462, –, –.
